# Re-Expression of *AKAP12* Inhibits Progression and Metastasis Potential of Colorectal Carcinoma *In Vivo* and *In Vitro*

**DOI:** 10.1371/journal.pone.0024015

**Published:** 2011-08-30

**Authors:** Weiwei Liu, Ming Guan, Tingting Hu, Xiaoye Gu, Yuan Lu

**Affiliations:** 1 Department of Laboratory Medicine, Huashan Hospital, Shanghai Medical College, Fudan University, Shanghai, People's Republic of China; 2 Central Laboratory, Huashan Hospital, Shanghai Medical College, Fudan University, Shanghai, People's Republic of China; Florida International University, United States of America

## Abstract

**Background:**

AKAP12/Gravin (A kinase anchor protein 12) is one of the A-kinase scaffold proteins and a potential tumor suppressor gene in human primary cancers. Our recent study demonstrated the highly recurrent loss of *AKAP12* in colorectal cancer and *AKAP12* reexpression inhibited proliferation and anchorage-independent growth in colorectal cancer cells, implicating *AKAP12* in colorectal cancer pathogenesis.

**Methods:**

To evaluate the effect of this gene on the progression and metastasis of colorectal cancer, we examined the impact of overexpressing *AKAP12* in the *AKAP12*-negative human colorectal cancer cell line LoVo, the single clone (LoVo-AKAP12) compared to mock-transfected cells (LoVo-CON).

**Results:**

pCMV6-AKAP12-mediated *AKAP12* re-expression induced apoptosis (3% to 12.7%, *p*<0.01), migration (89.6±7.5 cells to 31.0±4.1 cells, *p*<0.01) and invasion (82.7±5.2 cells to 24.7±3.3 cells, *p*<0.01) of LoVo cells *in vitro* compared to control cells. Nude mice injected with LoVo-AKAP12 cells had both significantly reduced tumor volume (*p*<0.01) and increased apoptosis compared to mice given AKAP12-CON. The quantitative human-specific Alu PCR analysis showed overexpression of *AKAP12* suppressed the number of intravasated cells in vivo (*p*<0.01).

**Conclusion:**

These results demonstrate that *AKAP12* may play an important role in tumor growth suppression and the survival of human colorectal cancer.

## Introduction

Colorectal cancer is the third most common form of cancer in the world and the leading cause of cancer mortality[1]–[3]. Better understanding of the mechanisms underlying colorectal cancer formation and progression is greatly needed because only modest improvements in the survival of colorectal cancer patients have been achieved over the last decade.

A-kinase anchor protein 12 (AKAP12/Gravin) was first isolated as a protein recognized by the serum of myasthenia gravis patients[4]. It is one of the A-kinase anchoring proteins (AKAPs) that belong to a family of scaffold proteins, and organizes the protein kinase A (PKA) and C (PKC)[5]. It is also an important regulator of the β_2_-adrenergic receptor complex, which controls cell signaling, cell adhesion, mitogenesis and differentiation[6], [7]. *AKAP12* has been mapped to chromosome 6q24–25.2, a cancer deletion hotspot [8]. DNA hypermethylation in the *AKAP12* promoter region, and the accompanied underexpression of the corresponding gene, has been noted in a variety of human cancers, including gastric cancer, esophageal cancer and lung cancer, and in myeloma cells and myeloid malignancies[6], [8]–[11]. Downregulation of *AKAP12* expression suggests that the inactivation of *AKAP12* expression may be linked to oncogenesis. A recent report using microarray data from *in silico* genetic searches indicated that methylation is associated with the downregulation of *AKAP12* in colon cancer and identified *AKAP12* as a potential tumor suppressor gene candidate[12]. In our previous study, downregulation or loss of *AKAP12* mRNA expression was detected in 68.9% (31/45) of colorectal carcinoma tissues and methylation of the *AKAP12* promoter region was detected in 77.8% (35/45) of these tissues compared with 13.3% (6/45) in the adjacent tissue[13]. Complete loss of *AKAP12* and hypermethylation of the promoter was detected in colorectal cancer cell lines, LoVo, SW480 and COLO320. However, it remains unclear if *AKAP12* plays an inhibitory role in the progression or metastasis of colorectal cancer.

In this report, we analyzed the effect of *AKAP12* on the tumorigenesis and metastasis of LoVo cells *in vitro* and *in vivo*. Our data demonstrate that the re-expression of *AKAP12* could inhibit the progression and metastatic potential of colorectal carcinoma. Therefore, we propose that *AKAP12* functions as a tumor suppressor of this solid cancer.

## Results

### AKAP12 inhibited cell growth and induced apoptosis

To examine the impact of the *AKAP12* re-expression in colorectal cancer cells, we constructed a vector constitutively expressing *AKAP12* (pCMV6-AKAP12) to restore *AKAP12* expression in LoVo cells, which lack *AKAP12* expression in our previous study[13]. We stably transfected LoVo cells with the pCMV6-AKAP12 or empty control vector, respectively. Significant *AKAP12* mRNA and protein re-expression in LoVo-AKAP12 cells compared with LoVo-CON was observed [13]. We also analyzed cell proliferation using a WST assay and the growth of LoVo cells in soft agar. Strongly inhibited the growth of LoVo cells and a significant decrease in the number of colonies was observed when tumor cells were transfected with pCMV6-AKAP12 [13].

**Figure 1:**
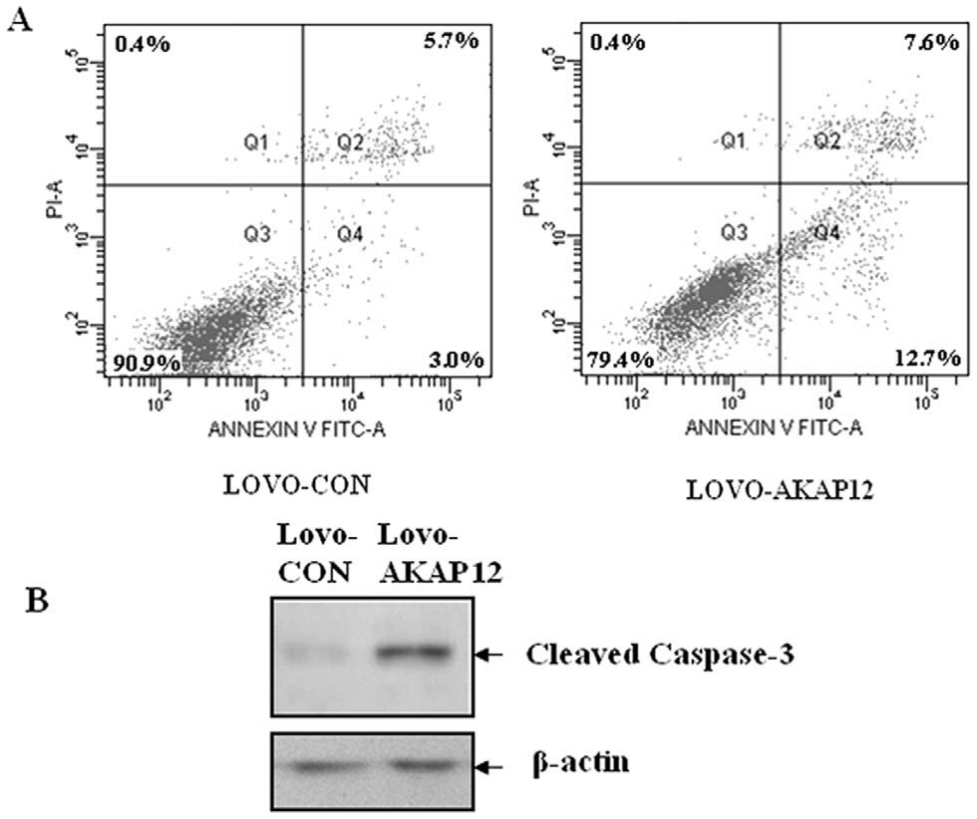
Effects of *AKAP12* on apoptosis of LoVo cells transfected with pCMV6-AKAP12. (A) Effects of *AKAP12* expression on apoptosis in AKAP12-transfected LoVo cells. LoVo-AKAP12 cells and LoVo-CON cells were analyzed using FACS and PI/Annexin V staining. The legends show the percentage of cells undergoing apoptosis. (B) Western blot analysis of cleaved-caspase-3 in Lovo-CON and Lovo-AKAP12 cells. AKAP12 increase cleaved-caspase-3 in cultured Lovo cells.

Given the effects of *AKAP12* on cell proliferation and viability, using PI and Annexin V staining, we next examined whether pCMV6-AKAP12 transfection-induced apoptosis of LoVo cells. Flow cytometry revealed that transfection of LoVo cells with pCMV6-AKAP12 induced a marked increased in the percentage of apoptotic (annexin V^+^ PI^−^) cells compared with the LoVo cells with control vectors (12.7% versus 3.0%, respectively; *p*<0.01) (Fig. 1A). We also evaluated the expression of cleaved-caspase-3 (the active form of caspase-3, Fig. 1B) in these two cells. The results indicate that cells transfected with the Lovo-AKAP12 can significantly increase cleaved-caspase-3 level.

### Oncosuppressive effect of AKAP12 on LoVo cells

**Figure 2:**
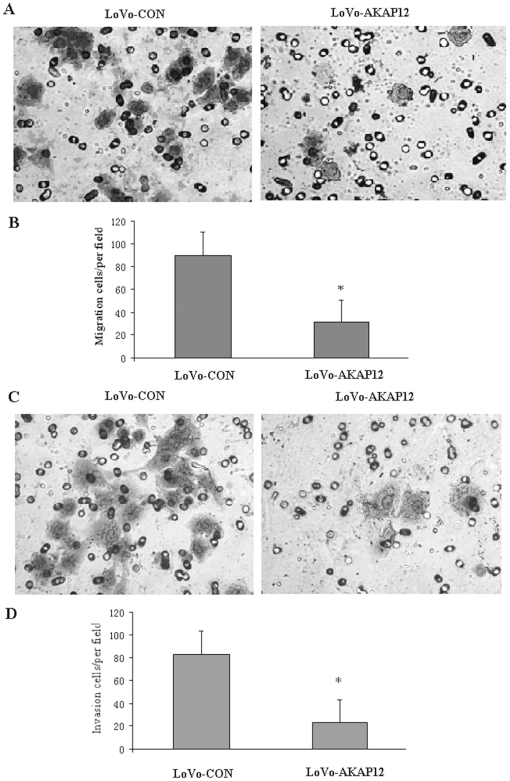
AKAKP12 suppresses *in vitro* migration and invasion ability of LoVo cells. (A) LoVo-AKAP12 and LoVo-CON cells penetrated through the transwell chambers and were photographed at ×100 magnification. (B) The *in vitro* migration ability of LoVo-AKAP12 cells and LoVo-CON cells was measured by determining the number of cells that migrated through the transwell chamber. Columns, mean values; bar, SD. * *p*<0.01. (C) LoVo-AKAP12 and LoVo-CON cells that migrated through the transwell chambers and photographed at ×100 magnification. (D) The *in vitro* migration ability of LoVo-AKAP12 cells and LoVo-CON cells was measured by determining the number of matrigel-coated cells that penetrated through the transwell chambers. Columns, mean values; bar, SD. *p<0.01.

Given that migration and invasion are key components of the metastatic cascade, we conducted *in vitro* migration and invasion assays to evaluate the migration and invasion potential of LoVo-AKAP12 and LoVo-CON cells. Overexpression of *AKAP12* lead to an average decrease in migration of an average of 65.4% compared to the control cells (31.0±4.1 versus 89.6±7.5 cells cells per field, respectively; *p*<0.01) (Fig. 2A, B). Furthermore, overexpression of *AKAP12* lead to an average of 70.1% (24.7±3.3 cells versus 82.7±5.2 cells per field, respectively; *p*<0.01) decrease invasiveness compared with the control cells (Fig. 2C, D). These results suggest that *AKAP12* expression is inversely associated with the migration and invasion ability of colorectal cell lines LoVo *in vitro*.

### AKAP12 inhibits tumorigenesis, metastatic and induced apoptosis in vivo

**Figure 3:**
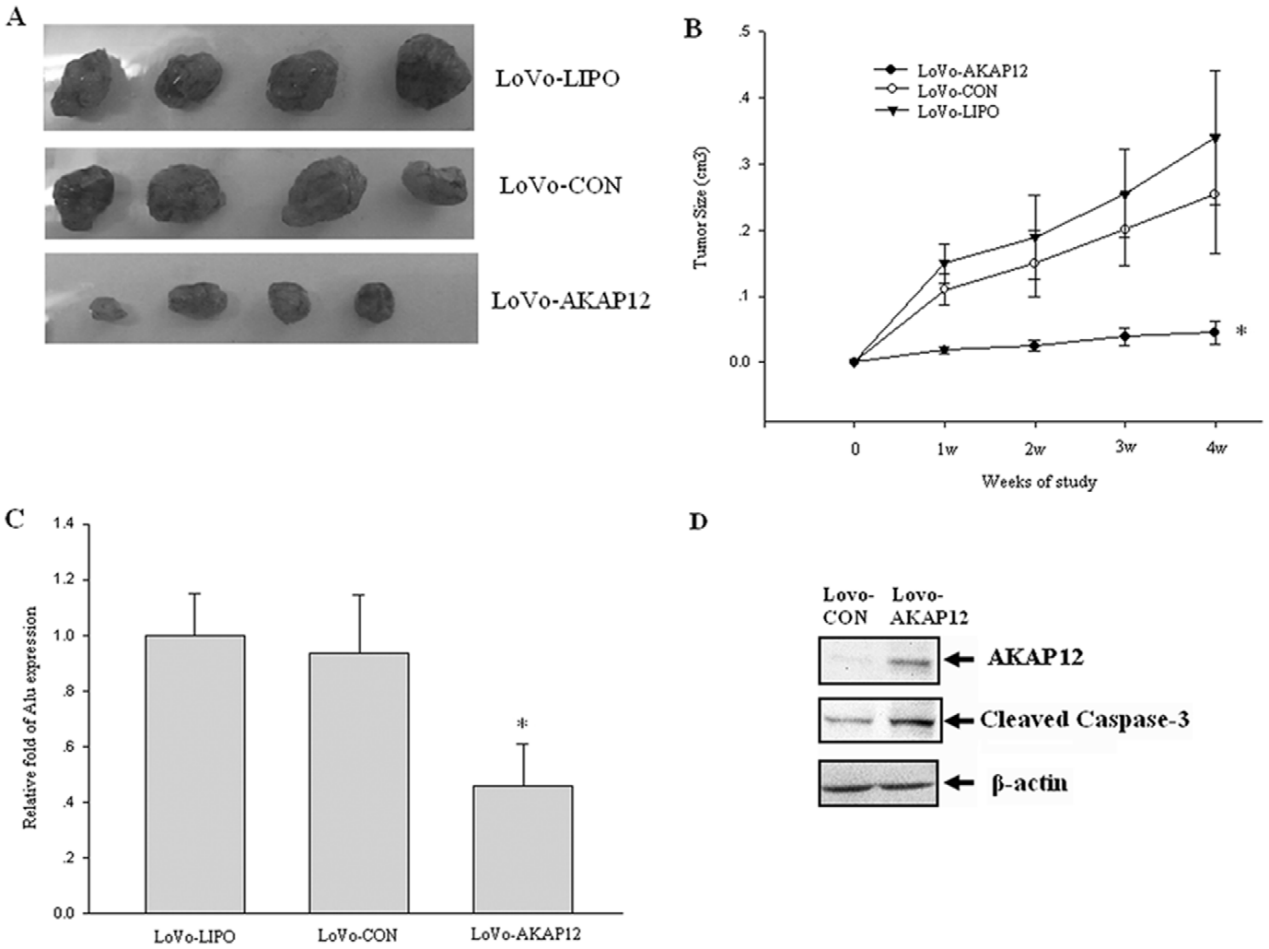
The effect of *AKAP12* on the growth of human primary xenografts. (A) *AKAP12* suppresses the growth of LoVo cells implanted in BALB/c nude mice. Mice injected with LoVo-AKAP12 cells displayed significantly reduced tumor volume compared with the animals injected with LoVo-CON or LoVo-LIPO cells four weeks after injection. (B) Tumor size was measured every week. Each point represents the mean volume ± SD on the X-axis. *p<0.01, LoVo-AKAP12 versus LoVo-CON versus LoVo-LIPO. *p*<0.01, LoVo-AKAP12 versus LoVo-CON. (C) Effect of *AKAP12* expression on the spontaneous metastasis of LoVo cells in nude mice. The quantitative real-time PCR amplification of the human Alu sequence and mouse *GAPDH* was performed using DNA extracts from the lung. (D) Western blot analysis of AKAP12 and cleaved-caspase-3 level in Lovo-CON and Lovo-AKAP12 group. AKAP12 and cleaved-caspase-3 were found to increase in the Lovo-AKAP12 group.

Because AKAP12 inhibits LoVo cell growth in vitro, we further assessed its effect on tumor formation in vivo. The anti-tumor activity of AKAP12 was evaluated in a primary xenograft model using nude mice. LoVo-AKAP12 cells, LoVo-CON cells and non-transfected LoVo-LIPO cells were injected into the flank of nude mice. Four weeks later, mice were sacrificed and examined for the presence of metastases in the lungs. Injection of LoVo-AKAP12 cells reduced tumor volume significantly compared with the LoVo-CON and LoVo-LIPO cells four weeks-post injection (p<0.01) (Fig. 3A, B). To assess if AKAP12 could reduce the formation of lung metastases, we determined the presence of Alu sequences in the lungs of nude mice injected with each type of LoVo cells using real-time PCR. The human tumor cell metastasis formation in mouse lung did not differ between LoVo-CON and LoVo-LIPO recipients. However, the formation of lung metastases in mice given LoVo-AKAP12 cells (6/10) was significantly lower than the mice receiving either LoVo-CON (10/10) or LoVo-LIPO (10/10) cells (Table 1). Additionally, lung tissue from mice injected with LoVo-AKAP12 cells displayed significantly less human Alu expression than LoVo-CON or LoVo-LIPO control group (Fig. 3C). We also evaluated the expression of AKAP12 and cleaved-caspase-3 level in the tumor tissues of LoVo-CON or LoVo-AKAP12 group (Fig. 3D). The results indicate that Lovo-AKAP12 group can significantly increase AKAP12 and cleaved-caspase-3 level. These data indicated that ectopic expression of AKAP12 in LoVo cells decreases their metastatic ability and induced apoptosis in vivo.

**Table 1:** Growth and metastatic ability of non-transfected and transfected LoVo cells *in vivo*.

Groups	Tumor		Lung metastases
	Incidence(growth/total)	Final tumor size, cm^3^±SD	Metastasis formation (metastasis/total)
LoVo-AKAP12	10/10	0.045±0.017*,**	6/10
LoVo-CON	10/10	0.254±0.089	10/10
LoVo-LIPO	10/10	0.34±0.102	10/10

*p<.01, LoVo-AKAP12 versus LoVo-CON and LoVo-LIPO.

**p<.01, LoVo-AKAP12 versus LoVo-CON.

## Discussion

The biological role of *AKAP12* in colorectal cancer progression and metastasis is poorly understood. In the present study, we examined this question through re-expression of *AKAP12* in *AKAP12*-null cancer cells to determine if this would influence cancer cell growth and metastatic potential. *AKAP12* inhibited cell growth, migration, invasion, anchorage-independent growth and induced apoptosis when transfected into LoVo cells. Furthermore, injection of LoVo-AKAP12, LoVo-CON or LoVo-LIPO cells into nude mice demonstrated that, *in vivo*, induced *AKAP12* expression in the colorectal cancer cells suppressed both tumor growth and metastasis and induced apoptosis.

Cancer develops when the balance between cell proliferation and cell death is disturbed, and aberrant cell proliferation leads to tumor growth. In human gastric cancer cells, re-expression of *AKAP12* lead to reduced colony formation and apoptotic cell death[8]. To examine whether modulation of *AKAP12* expression influenced the tumorigenic properties of the colorectal cancer cells, we tested in vitro LoVo cell proliferation and apoptosis. As shown by both the WST assay and FACS analysis, *AKAP12* displays tumor-suppressive activity in vitro. AKAP12-mediated suppression of tumor growth may be achieved through direct or indirect interaction with multiple apoptotic proteins and associated signaling pathways in cancer cells[14]. These include several signaling molecules that participate in cell proliferation and cytoskeletal organization, as well as protein kinase C (PKC), protein kinase A (PKA), cyclin D1 and calmodulin[15]–[17]. In HT1080 cells, *AKAP12* was found to suppress tumor cell viability by inducing apoptosis via caspase-3 and was associated with a decreased expression of Bcl-2 and increased expression of Bax[18]. Moreover, *AKAP12* induced the expression of Cip1/p21 and Kip1/p27 and decreased the expression of cyclin D1[18]. SSeCKS, is the orthologue of human *AKAP12* gene, re-expression was reported to result in the attenuation of critical Src-induced proliferative and pro-angiogenic gene expression, including Afp and Cdc20a, and cell cycle regulatory genes such as Ptpn11 and Gadd45a[19].

Cancer cells are capable of anchorage-independent growth and are able to travel and settle in a new site in which the microenvironment may be completely different[20], [21], a process crucial for oncogenesis and cancer metastasis. The re-expression of *AKAP12* in LoVo cells reduced colony formation and inhibited anchorage-independent growth ability, which may explain the contribution of *AKAP12* to colorectal cancer progression. However, in this study single clone data of a single cell line may be could not describe the exact role of AKAP12 in the progression of colorectal cancer and needs further exploration.

Distant metastases are the major cause of morbidity and mortality in human colorectal cancer. *AKAP12* may inhibit early steps in metastatic colonization[22]. SSeCKS has been reported to suppress lung metastasis of MatLyLu prostate cancer cells, correlating with its suppression of VEGF–165 and -121 isoforms[23]. They also found that SSeCKS suppresses serum-induced activation of the Raf/MEK/ERK pathway and downregulates matrix metalloproteinase (MMP)-2/9 expression and secretion[24]. It also has been proposed that AKAP12/Gravin functions as an essential scaffold for regulatory proteins that suppress the migratory behavior of the mesoderm during gastrulation in zebrafish embryos[25]. It has further been suggested that this function explains how *AKAP12* inhibits the invasive behaviors of metastatic cells. In agreement with this hypothesis, our results demonstrated that, *in vitro,* induced expression of *AKAP12* could reduce the invasion or migration ability of oncogenic cells and significantly suppress the metastatic potential of the colorectal cancer cells. *AKAP12* also could inhibit colorectal cancer tumorgenesis and metastasis *in vivo* and suggests that *AKAP12* may hold great promise for designing novel therapeutic strategies against this solid cancer.

Our study indicates that *AKAP12* is a suppressive factor in colorectal cancer cells, inhibiting tumor cell growth and metastases *in vitro* and *in vivo.* Collectively, these findings demonstrated that *AKAP12* may play an important role in the development of malignancies. Therefore, *AKAP12* could serve as an effective target for the development of novel anti-cancer therapies and may be a useful biomarker for human colorectal cancer.

## Materials and Methods

### Cell lines

The colorectal carcinoma cell line, LoVo, was obtained from the American Type Culture Collection (ATCC, Manassas, VA, USA). Cells were cultured in F12K media (GibcoBRL, Grand Island, NY, USA) supplemented with 10% fetal bovine serum (Sigma, St. Louis, MO, USA) and penicillin-streptomycin (GibcoBRL). Cells were incubated in 5% CO_2_ at 37°C, and only cells with a passage number of <10 were used in the experiments.

### Recombinant vectors and stable transfection

pCMV6-XL4-AKAP12 (Cat No. SC110078) was purchased from Origene (Rockville, MD). The plasmids were purified, digested with *NotI* and subcloned into the pCMV6-Neo expression vector. LoVo cells were transfected with the pCMV6-Neo-AKAP12 construct (LoVo-AKAP12), or the control empty vector (LoVo-CON). For stable transfections, 10 µg of plasmid DNA was resuspended in 500 µl of serum-free DMEM and mixed with 60 µl Lipofectamine 2000 (Invitrogen, Carlsbad, CA, USA) in 500 µl of serum-free DMEM. The mixture was incubated for 20 min at room temperature and added to a 70%-80% confluent 100-mm tissue culture dishes. After 2 h, the transfection medium was changed to F12K medium containing 10% FBS. Transfection efficiency was typically 60–80% as determined by GFP expression. After 48 h, the transfection medium was changed to F12K medium containing 10% FBS and antibiotic geneticin (G418) (400 µg/ml) (Invitrogen Life Technologies, Inc., Carlsbad, CA). Clones were screened by limited dilution cloning 4 days after selection. *AKAP12* expression levels using Western blots, and one clone with vector alone (LoVo-CON) and a clone overexpressing *AKAP12* (LoVo-AKAP12) were selected for further experiments.

### Flow cytometry assay

Analysis of apoptosis was determined by flow cytometry. LoVo-AKAP12 and LoVo-CON cells were seeded in six-well plates in 10% FBS/F12K and then incubated at 37°C. FACS analysis using propidium iodide (PI) staining combined with Annexin V-FITC (BD, Franklin Lakes, NJ, USA) was performed. First, 10^6^ cells were washed twice with cold PBS and resuspended in 1× binding buffer. Then, 10^5^ cells were transferred to a 5 mL tube, 5 µL of Annexin V-FITC and PI were added and the cells were incubated in the dark for 15 min at room temperature. Prior to FACS analysis, 400 µL of 1× binding buffer were added. The experiment was repeated three times.

### Western blot analysis

Western blot analysis was performed as described previously[26]. Equala mounts of proteins from cells and tissues were separated by SDS-PAGE gels and then transferred to PVDF membranes. After blocking, the membranes were incubated with the appropriate primary antibodies overnight (anti-cleaved-caspase-3, 1∶500, Cell Signaling; anti-β-actin, 1∶500, Cell Signaling; anti-human AKAP12, 1∶500, Sigma). Following three washes with TBST, the blots were incubated with the secondary horseradish peroxidase-conjugated antibody (1∶3000) at room temperature for 1 h. Immunocomplexes were visualized by using enhanced chemiluminescence (BioRad) following the manufacturer's instructions. The bands were subsequently analyzed densitometrically with Quantity One Software (BioRad).

### In vitro cell migration and invasion assay

Cell migration was evaluated using the QCM 24-Well Colorimetric Cell Migration Assay Kit (Chemicon). In brief, LoVo-AKAP12 and LoVo-CON cells were plated at a density of 1×106 cells/mL on 8 µm inserts and cultured for 24 h. The non-migrating cells were removed from the upper surface of the insert with a cotton-tipped swab, and cells that have migrated are incubated with stain solution. Transfer the stained insert to a clean well containing 200 µL of Extraction Buffer for 15 minutes at room temperature. Extract the stain from the underside by gently tilting the insert back and forth several times during incubation. Remove the insert from the well. Then the insert could be counted manually through a microscope and five fields were evaluated for migration. The experiment was repeated three times.

The invasive capability of the transfected cells was assessed using Cell Invasion Assay Kit (Chemicon), a similar chemotaxis chamber as above but with a few modifications. In brief, LoVo-AKAP12 and LoVo-CON cells were plated at a density of 10^6^ cells/mL on ECMatrix gel-coated polycarbonate membrane inserts with an 8 µm pore size and cultured for 24 h. Invasive cells migrate through the ECM layer and cling to the bottom of the polycarbonate membrane. The non-invading cells and the ECMatrix gel were removed from the upper surface of the insert with a cotton-tipped swab. Add staining solution to the unoccupied wells of the plate and stain invasive cells on lower surface of the membrane by dipping inserts in the staining solution. Dip inserts in a beaker of water several times to rinse. Count cells by photographing the membrane through the microscope and five fields were evaluated for invading cells. The experiment was repeated three times.

### Primary xenografts

To evaluate in vivo tumorigenesis, a colorectal cancer xenografting mouse model was used. Female BALB/c nude mice of four to six weeks old were prepared for tumor implantation. Mice were allowed to acclimate for one week after arrival. All animals were maintained in a sterile environment on a daily 12-h light/dark cycle. After resuspension in PBS, LoVo-AKAP12, LoVo-CON cells and LoVo-LIPO (transfected with Lipofectmine only), 5×106 cells/mouse were injected subcutaneously into the flank of nude mice (n = 10/group). Tumor volume was calculated weekly for four weeks according to the formula, TV (cm3)  =  length × width2×0.5. The primary xenografts and lungs were harvested, weighed, and snap-frozen[27]. All of the animal experiments were conducted in strict accordance with the National Institute of Health Guide for the Care and Use of Laboratory Animals.

### Metastasis assay in vivo

The detection of the presence of human tumor cells in the lungs of mice was achieved using the quantitative detection of human Alu sequences present in total lung genomic DNA preparations[28]. Whole lungs were harvested and genomic DNA was isolated using the DNeasy Tissue kit (Qiagen). Lung genomic DNA was quantified in human tumor cells from the lungs using a PCR-based detection of the human Alu sequences and, as a control, GAPDH, and the following primers: Alu-F, 5′-CACCTGTAATCCCAGCACTTT-3′; Alu-R, 5′-CCCAGGCTGGAGTGCAGT-3′; GAPDH-F, 5′- GCACAGTCAAGGCCGAGAAT-3′and GAPDH-R,5′- GCCTTCTCCATGGTGGTGAA-3′. PCR was performed under the following conditions: 95°C for 2 min, 30 cycles at 95°C for 30 s, 65°C for 20 s, and 72°C for 20 s. The SYBR Green PCR Master Mix (Qiagen) was used for the real-time PCR amplification of the Alu sequences. A quantitative measure of amplifiable mouse DNA was obtained through amplification of the mouse GAPDH genomic DNA sequence. The fluoresence emitted by the reporter dye was detected using the SYBR Green and the thershold cycle (Ct) for each sample was recorded as a quantitative measure of the amount of product in each sample. When indicated, the Alu signal was normalized against the relative quantity of GAPDH and expressed as ΔCt = (Ct^Alu^–Ct^GAPDH^). The changes in Alu signal relative to the total amount of genomic DNA were expressed as 2^−−ΔCt^, indicating the changes in the quantity of human DNA in the mouse lung tissue. Data are shown as relative to the expression of Alu and levels from the LoVo-LIPO group were normalized to one. The specificity of human Alu PCR was verified by the lack of amplification from 100% mouse DNA.

### Statistical analysis

The Student's t-test was used to compare the obtained values with those from the corresponding control experiments and p-values <.05 was considered statistically significant.

### Ethical Treatment of Animals

This study was carried out in strict accordance with the recommendations in the relevant national and international guidelines for the Care and Use of Laboratory Animal. The protocol was approved by the Committee on the Ethics of Animal Experiments of the Department of Laboratory Animal Science, Shanghai Jiao Tong University School Of Medicine (Permit Number: 2009014). All efforts were made to minimize suffering.

## References

[1] Wallner M, Herbst A, Behrens A, Crispin A, Stieber P (2006). Methylation of serum DNA is an independent prognostic marker in colorectal cancer.. Clin Cancer Res.

[2] Agrawal A, Murphy RF, Agrawal DK (2007). DNA methylation in breast and colorectal cancers.. Mod Pathol.

[3] Jemal A, Siegel R, Ward E, Murray T, Xu J (2007). Cancer statistics 2007.. CA Cancer J Clin.

[4] Gordon T, Grove B, Loftus JC, O'Toole T, McMillan R (1992). Molecular cloning and preliminary characterization of a novel cytoplasmic antigen recognized by myasthenia gravis sera.. J Clin Invest.

[5] Nauert JB, Klauck TM, Langeberg LK, Scott JD (1997). Gravin, an autoantigen recognized by serum from myasthenia gravis patients, is a kinase scaffold protein.. Curr Biol.

[6] Tessema M, Willink R, Do K, Yu YY, Yu W (2008). Promoter methylation of genes in and around the candidate lung cancer susceptibility locus 6q23-25.. Cancer Res.

[7] Wang HY, Tao J, Shumay E, Malbon CC (2006). G-Protein-coupled receptor-associated A-kinase anchoring proteins: AKAP79 and AKAP250 (gravin).. Eur J Cell Biol.

[8] Choi MC, Jong HS, Kim TY, Song SH, Lee DS (2004). AKAP12/Gravin is inactivated by epigenetic mechanism in human gastric carcinoma and shows growth suppressor activity.. Oncogene.

[9] Jin Z, Hamilton JP, Yang J, Mori Y, Olaru A (2008). Hypermethylation of the AKAP12 promoter is a biomarker of Barrett's-associated esophageal neoplastic progression.. Cancer Epidemiol Biomarkers Prev.

[10] Flotho C, Paulun A, Batz C, Niemeyer CM (2007). AKAP12, a gene with tumour suppressor properties, is a target of promoter DNA methylation in childhood myeloid malignancies.. Br J Haematol.

[11] Heller G, Schmidt WM, Ziegler B, Holzer S, Mullauer L (2008). Genome-wide transcriptional response to 5-aza-2′-deoxycytidine and trichostatin a in multiple myeloma cells.. Cancer Res.

[12] Mori Y, Cai K, Cheng Y, Wang S, Paun B (2006). A genome-wide search identifies epigenetic silencing of somatostatin, tachykinin-1, and 5 other genes in colon cancer.. Gastroenterology.

[13] Liu W, Guan M, Su B, Ye C, Li J (2010). Quantitative assessment of AKAP12 promoter methylation in colorectal cancer using methylation-sensitive high resolution melting: Correlation with dukes' stage.. Cancer Biol Ther.

[14] Lowe SW, Lin AW (2000). Apoptosis in cancer.. Carcinogenesis.

[15] Lin X, Nelson P, Gelman IH (2000). SSeCKS, a major protein kinase C substrate with tumor suppressor activity, regulates G(1)-->S progression by controlling the expression and cellular compartmentalization of cyclin D.. Mol Cell Biol.

[16] Lin X, Gelman IH (1997). Reexpression of the major protein kinase C substrate, SSeCKS, suppresses v-src-induced morphological transformation and tumorigenesis.. Cancer Res.

[17] Xia W, Unger P, Miller L, Nelson J, Gelman IH (2001). The Src-suppressed C kinase substrate, SSeCKS, is a potential metastasis inhibitor in prostate cancer.. Cancer Res.

[18] Yoon DK, Jeong CH, Jun HO, Chun KH, Cha JH (2007). AKAP12 induces apoptotic cell death in human fibrosarcoma cells by regulating CDKI-cyclin D1 and caspase-3 activity.. Cancer Lett.

[19] Liu Y, Gao L, Gelman IH (2006). SSeCKS/Gravin/AKAP12 attenuates expression of proliferative and angiogenic genes during suppression of v-Src-induced oncogenesis.. BMC Cancer.

[20] Frisch SM, Francis H (1994). Disruption of epithelial cell-matrix interactions induces apoptosis.. J Cell Biol.

[21] Liotta LA, Kohn E (2004). Anoikis: cancer and the homeless cell.. Nature.

[22] Li HZ, Gao Y, Zhao XL, Liu YX, Sun BC (2009). Effects of Raf Kinase Inhibitor Protein Expression on Metastasis and Progression of Human Breast Cancer.. Mol Cancer Res.

[23] Su B, Zheng Q, Vaughan MM, Bu Y, Gelman IH (2006). SSeCKS metastasis-suppressing activity in MatLyLu prostate cancer cells correlates with vascular endothelial growth factor inhibition.. Cancer Res.

[24] Su B, Bu Y, Engelberg D, Gelman IH (2009). SSeCKS/Gravin/AKAP12 inhibits cancer cell invasiveness and chemotaxis by suppressing a PKC-RAF/MEK/ERK pathway.. J Biol Chem.

[25] Weiser DC, Pyati UJ, Kimelman D (2007). Gravin regulates mesodermal cell behavior changes required for axis elongation during zebrafish gastrulation.. Genes Dev.

[26] Guan M, Yam HF, Su B, Chan KP, Pang CP (2003). Loss of pigment epithelium derived factor expression in glioma progression.. J Clin Pathol.

[27] Li J, Huang H, Sun L, Yang M, Pan C (2009). MiR-21 indicates poor prognosis in tongue squamous cell carcinomas as an apoptosis inhibitor.. Clin Cancer Res.

[28] Lee WJ, Chen WK, Wang CJ, Lin WL, Tseng TH (2008). Apigenin inhibits HGF-promoted invasive growth and metastasis involving blocking PI3K/Akt pathway and beta 4 integrin function in MDA-MB-231 breast cancer cells.. Toxicol Appl Pharmacol.

